# Perceptions of Post-Stroke Fatigue among Health Care Practitioners: A Qualitative Interview Study

**DOI:** 10.3390/medicina59122146

**Published:** 2023-12-11

**Authors:** Wafa Saeed Alahmari, Reem M. Basuodan, Kholood Matouq Shalabi, Ahmed Saad Alhowimel, Mazyad Alotaibi, Eirini Kontou, Pip Logan, Neil Coulson

**Affiliations:** 1 Department of Rehabilitation Sciences, College of Health and Rehabilitation Sciences, Princess Nourah bint Abdulrahman University, P.O. Box 84428, Riyadh 11671, Saudi Arabia; wsalahmari@pnu.edu.sa (W.S.A.); kmshalabi@pnu.edu.sa (K.M.S.); 2 Department of Health and Rehabilitation Sciences, College of Applied Medical Sciences, Prince Sattam bin Abdulaziz University, P.O. Box 84428, Alkharj 11671, Saudi Arabia; a.alhowimel@psau.edu.sa (A.S.A.); maz.alotaibi@psau.edu.sa (M.A.); 3 Mental Health & Clinical Neurosciences, School of Medicine, University of Nottingham, Jubilee Campus, Nottingham NG7 2TU, UK; eirini.kontou@nottingham.ac.uk; 4 Institute of Mental Health, Nottinghamshire Healthcare NHS Foundation Trust, University of Nottingham, Jubilee Campus, Nottingham NG7 2TU, UK; 5 Centre for Rehabilitation and Ageing Research, School of Medicine, University of Nottingham, Sutton Bonington LE12 5RD, UK; pip.logan@nottingham.ac.uk; 6 Nottingham Centre for Public Health and Epidemiology, School of Medicine, University of Nottingham, Sutton Bonington LE12 5RD, UK; neil.coulson@nottingham.ac.uk

**Keywords:** stroke, fatigue management, occupational therapists, physiatrists, physiotherapists

## Abstract

*Background and Objectives*: Post-Stroke Fatigue (PSF) is a complex, multidimensional, debilitating condition that affects almost half of all stroke survivors. This study explored the perceptions of physiatrists, physiotherapists, and occupational therapists about PSF and their experiences in managing patients with PSF in Saudi Arabia. *Materials and Methods*: Qualitative semi-structured interviews were conducted with participants from three different groups: eight physiotherapists (PTs), eight occupational therapists (OTs), and eight physiatrists (DRs). Using purposive sampling, participants with at least one year of experience in the field of PSF management were invited to take part. The data were analysed using inductive thematic analysis. *Results:* Twenty-four health care participants (eight PTs, eight OTs, eight DRs) were recruited. Five overarching themes encompassing various subthemes and sub-subthemes were generated: ‘knowledge about post-stroke fatigue’, ‘diagnosing post-stroke fatigue’, ‘treatment approach’, ‘lack of awareness about post-stroke fatigue’, and ‘domains to improve’. The data indicated that participants used various strategies to manage PSF, including dietary changes, sleep hygiene, exercise, and energy conservation. *Conclusions*: Participants acknowledged that they lacked PSF-related management skills, despite possessing adequate knowledge about the management of stroke. Their openness to participating in activities that would improve their ability to diagnose and manage PSF was particularly striking.

## 1. Introduction

Post-Stroke Fatigue (PSF) is a complex, multidimensional, debilitating condition that affects almost half of all stroke survivors and is one of the most frustrating residual outcomes for stroke patients [1]. It is the result of as-yet poorly understood and complex interactions across psychosocial, behavioural, and biological phenomena [2]. Various definitions of PSF have been proposed; one of them, for example, defines it as a condition affecting normal activities due to insufficient physical energy [3]. PSF is also considered as an independent predictor of long-term disability, inability to perform daily activities, and increased burden of care for caregivers [4].

Patients often perceive PSF to be a result of gaps in the management of their condition by health care practitioners (HCPs) [5]. For example, 40% of individuals with PSF reported that they did not receive any kind of treatment or support [6], and almost 84% felt that their long-term needs were not being satisfactorily met [7]. This situation is compounded by the lack of effective treatment and management strategies and insufficient information about the aetiology of the condition [8,9,10]. Thus, it is important to understand the causative mechanisms and identify at-risk patients. 

There is a scarcity of literature about PSF for Saudi Arabian HCPs, which have a diverse population and healthcare system. Saudi Arabia’s Ministry of Health provides health care services at three levels; primary, secondary, and tertiary care [11]. Physical therapists, occupational therapists, and physiatrists are available in secondary- and tertiary-level hospitals, which is why this study chose these three groups of health professionals. There is also a lack of evidence around effective practices in identifying and managing PSF in Saudi Arabia. For a better understanding of the appropriate treatment and management modalities in the context of Saudi Arabia, it is important to explore stroke survivors’ and HCPs’ perceptions of PSF. Accordingly, the main aim of this study was to explore current perceptions and experiences related to the understanding and management of PSF among HCPs including physiatrists (physicians who specialise in rehabilitation) (DRs), occupational therapists (OTs), and physiotherapists (PTs) in Saudi Arabia.

## 2. Materials and Methods

### 2.1. Sampling Method

The maximum variation method of purposive sampling was used to recruit the most suitable study participants [12] and ensure participant diversity in terms of their contributions to the answers to the research questions. This study also used a small but representative sample to record a broad spectrum of perspectives [12]. PTs, DRs, and OTs who have experience in providing rehabilitation care to stroke survivors were included, with male and female participants within each group. The maximum variation method was employed in this study to capture the broadest possible range of experiences associated with PSF. This diversity of experiences was crucial to the study as it enabled the researchers to identify patterns and themes that might have eluded detection if a more homogeneous sample had been employed. The research questions delved into the experiences of PSF among individuals from diverse backgrounds, encompassing various specializations, levels of experience (junior, senior), genders, ages, and qualification levels (postgraduate to bachelor’s degree).

### 2.2. Recruitment

The primary researcher (WA) explained the purpose and inclusion criteria of the study to the rehabilitation unit head of King Fahad Medical City Hospital in Saudi Arabia to select PTs, DRs, and OTs in his staff who met the study’s participant-inclusion criteria and had an interest in participating. Then, the primary researcher interviewed participants who met the inclusion criteria and had expressed initial interest in participating. A mutually convenient date and venue were arranged. All the interviews were conducted in person by the primary researcher. Before each interview, all interviewees were given an information sheet to read and an informed consent form to sign assuring them that they were free to withdraw at any point and that their data would be anonymised before the interview.

### 2.3. Ethical Considerations

This study was approved by the Faculty of Medicine and Health Sciences Ethics Committee of the University of Nottingham (Ethics No. 2631603, 6 September 2019). Ethical approval was obtained from the hospital of King Fahad Medical City (IRB No. 00010471).

### 2.4. Interviews

The interviews were semi-structured and included pre-defined open-ended questions based on the aims of the study (Table 1) and based on the gap highlighted in the previous literature [9]. In addition to key information about PSF, data on sociodemographic characteristics, including their sex, qualification, occupational status, and type and years of experience, were also collected. The interview guide was piloted with two HCPs and some words in the questions were changed, such as reworded and simplified. The primary researcher (WA), who conducted the interviews, used such open-ended questions as leads and used prompts to delve deeper into the participants’ responses. The interviews were held in a quiet and comfortable meeting room within the King Fahad Medical City Hospital premises. A digital recorder was used to record the interviews, which lasted for 30–60 min. Although the HCPs were proficient in the English language, the interviews were conducted in their mother tongue (Arabic) as the researcher deemed that they would feel more comfortable discussing their perceptions and experiences of PSF in Arabic. While conducting the interviews, the researcher (WA) observed data saturation after interviewing around 20 participants, as the same codes and themes were repeatedly found in the latter interviews. However, since the participants were already shortlisted and their time was booked for the interviews, the rest of the interviews were also conducted.

### 2.5. Data Analysis

Interview data were transcribed verbatim into Arabic transcripts. WA, who is a native Arabic speaker, translated the transcripts into English. Then, the translated transcript was validated by comparing it to English translation drafts by two other researchers, and a final translated English draft was agreed upon. Interview data were transcribed verbatim into Arabic transcripts via a two-way process of translation and re-translation by various unrelated personnel to validate the qualitative data and the translation process. One native Arabic-speaking researcher translated the transcripts into English and then validated them by comparing them to English translation drafts prepared by two other researchers. This draft was translated into Arabic by two members of the department and by two researchers to compare with the original Arabic transcripts. All four researchers involved in the translation process were doctoral-level researchers in the Department of Orthopaedic Physiotherapy, were proficient in both English and Arabic, and were not involved in the research in any other way. This two-way process of translation and re-translation by various unrelated personnel was performed to validate the qualitative data and the translation process. Then, an inductive thematic analysis of the interview transcripts was carried out [13], and codes and themes were assigned to the respective transcripts by WA. 

To improve the study’s credibility and internal validity, WA, who has both academic qualifications and clinical experience with stroke, carefully examined the study for any source of potential bias. Prior to the interviews, WA made efforts to ensure that the participants understood the study purpose and processes and how their information would be used as building blocks for further research and how it could improve PSF patient care. In order to avoid researcher-induced selection bias, maximum variation of purposive sampling was conducted by a department coordinator who was not a researcher, while all study researchers were blinded to the sample selection process. The research design, participant recruitment, themes of the interviewees’ responses, and data analysis methods were discussed and agreed upon by all the researchers to prevent bias and to ensure the reliability and reproducibility of the findings. The themes of the interviewees’ responses and the adopted methods were discussed and agreed upon by all the researchers to prevent bias. The research design, participant recruitment, and data analysis methods were finalised after extensive discussion with the research supervising team to ensure the reliability and reproducibility of the findings. 

The six key phases of thematic analysis by [13] Braun and Clarke (2006) for analysing the interview data were incorporated with the recommended means of establishing trustworthiness [14]. These phases are: getting acquainted with the data, creating initial codes, looking for themes, reviewing the identified themes, defining and naming the themes, and finally, generating a report. This qualitative study was developed following these key stages as the primary researcher got acquainted with the interview transcripts, created the initial codes, generated the themes, and defined and named them. The data were analysed without trying to fit them into an existing coding frame or into the researcher’s pre-conceived notions, impressions, and ideas. The primary researcher thoroughly read the interview transcripts and identified relevant codes. Once a set of similar codes was identified, the codes were assigned to broader themes. Then, the primary researcher conducted the initial coding and mapping according to appropriate sub-themes and themes. Then, the research team met and provided their feedback on this process of inductive thematic analysis and the codes and themes were refined based on this feedback. The research team agreed upon the final stages of this mapping exercise, and collectively decided to group codes into respective themes. The final thematic framework was then discussed and approved by the supervisory team.

## 3. Results

### 3.1. Study Participants

This study included 24 participants, including physiotherapists (n = 8), occupational therapists (n = 8), and physiatrists (n = 8) who had 2 to 20 years of experience in handling stroke survivors (mean = 9.04 years, SD = 5.08).

### 3.2. Themes and Subthemes

Five major themes with several subthemes under each were generated (Table 2).

#### 3.2.1. Theme 1: Knowledge about Post-Stroke Fatigue

**Demographic, clinical, and lifestyle factors.** Most participants believed that PSF was caused by and associated with multiple factors such as outcomes of the patient’s past medical history, lifestyle, physical impairment, activity, level of endurance, and demographic characteristics.

*‘Also, fatigue after a stroke depends on the patient’s lifestyle before the stroke, age and past medical history. All these factors affect the severity of fatigue after stroke…The physical activity level before a stroke affects or reflects the severity of the fatigue after the stroke.’ (PT2)*

*‘Actually, my only idea about fatigued stroke survivors is that they usually have low endurance.’ (OT4)*

Several respondents believed that specific demographic characteristics, such as gender and age, and the presence of comorbidities, such as diabetes, were associated with an increased incidence or severity of PSF.

*‘First of all, stroke patients will have PSF when they have other comorbidities, such as diabetes.’ (DR2)*

**Psychological factors.** The participants perceived mutual interactions between PSF and psychological factors, including depression, fear, and cognitive impairment. Several participants strongly felt that depression in stroke survivors resulted in PSF. Further, PSF symptoms were considered to be aggravated by cognitive impairments, which were linked to depression. This places an additional cognitive burden on patients and might lead to deficits in social functioning and interpersonal communication.

*‘The most common cause of PSF is depression. Patients with depression have the highest risk of developing fatigue.’ (DR6)*

*‘Also, patients’ depression, communication problems and lack of cognitive involvement will cause more fatigue.’ (PT7)*

#### 3.2.2. Theme 2: Diagnosing Post-Stroke Fatigue

**Assessing endurance levels**. The PTs believed fatigue levels could be used as a proxy for the endurance levels of stroke survivors.

*‘I measure the fatigue or endurance level by the ability of patients to walk for more than 6 min without rest. In this case, it means he has a good endurance level and does not have fatigue.’ (PT8)*

They also drew attention to their understanding of the relationships between fatigue, endurance, and functional levels.

*‘I can measure the fatigue level in correlation with the endurance and functional levels.’ (PT7)*

**Assessing functional levels.** Most participants reported that they estimated PSF in stroke survivors by assessing their functional ability and dependency levels instead of using available valid PSF assessment tools.

*‘If a patient has a low functional independence level or functional independence score, of course he or she has more fatigue. Conversely, if a patient has a high functional independence level or functional independence score, of course he or she has less fatigue.’ (OT8)*

#### 3.2.3. Theme 3: Treatment Approaches

The majority of PTs focused on exercises, group exercise, and endurance training; the majority of OTs talked about energy conservation, simplification of work, and prioritization of tasks, while the DRs mainly focused on managing confounding factors such as diabetes, hypertension, urinary tract infection, depression, dietary habits, and sleep hygiene.

**Managing confounding factors**. The HCPs believed that, to effectively treat PSF, it was important to determine which symptoms were being caused by other comorbidities such as diabetes mellitus, vitamin deficiencies, and infections. Some other factors that were considered to contribute to PSF included nutritional deficiencies and inadequate hydration.

*‘We have to do some lab tests, such as the thyroid function test, complete the blood screening for haemoglobin, irons. You have to check for irons and vitamin D.’ (DR4)*

*‘The patients may not have sufficient nutrition and hydration. They need these to be able to meet their energy demand; not having these will have an impact on their fatigue.’ (DR6)*

**Sleep management**. The interviewees viewed sleep management as an important component of PSF management. They highlighted the importance of monitoring sleep via targeted therapies and medications to mitigate the negative effects of PSF to some extent.

*‘First, we’ll ask and talk in detail about sleep hygiene, where problems are very common. Post-stroke survivors often have a sleep pattern issue.’ (DR1)*

*‘We monitor their sleep. If the sleep pattern is not good, the patient gets fatigued next time. We give some sleep medications until we achieve rhythm, regular rhythm.’ (DR3)*

**Exercise therapy.** Several interviewees considered exercise therapy to be helpful in the treatment of PSF. They provided several examples of exercise therapy, such as, for example, gradual endurance training, breathing, endurance, exercises and group exercise sessions, and allowing adequate rest periods between exercises.

Endurance and breathing exercises: Several interviewees also highlighted the importance of doing exercises gradually, with adequate time for rest in between exercises according to their experiences.

The specific types of endurance exercises included long-distance outdoor walking, biking, aerobics, and treadmill exercises. Another participant stated that the main focal points for improving patients’ wellbeing via endurance exercises were endurance, gait, and balance, which were viewed as effective focal points for alleviating PSF.

*‘I usually recommend and teach them [fatigue stroke survivors] breathing exercises.’ (OT5)*

*‘Gradual training and long-distance outdoor walking such as shopping or going for any kind of outdoor activity are effective.’ (PT5)*

*‘During physiotherapy sessions, we focus on treadmill, bike and aerobic exercise, gradually increasing the patient’s fitness level.’ (OT3)*

*‘Group aerobic exercises for stroke survivors with similar functional levels may be helpful in decreasing fatigue symptoms.’ (PT3)*

Several therapists also stated that breathing exercises were effective for reducing PSF.

Group exercises: Several participants described the use of group exercise sessions in the management of stroke survivors.

*‘We use rest between exercises, gradual exercises and functional training.’ (PT4)*

**Energy conservation techniques.** The participants mentioned the importance of teaching patients how to use energy conservation methods, such as work simplification, napping, and assistive devices, to lessen and manage their PSF.

*‘I give fatigued stroke survivors some instructions, such as energy conservation and work simplification.’ (OT2)*

*‘The other advice I give to my patients is to take a nap in the afternoon.’ (DR1)*

*‘Sometimes I give instructions for stroke survivors to use assistive devices in walking long distances to avoid easily getting fatigued.’ (PT4)*

**Educating patients and families.** The education of both patients and their caregivers/families about PSF was considered to be beneficial by the participants.

*‘I think home education is one of the battled factors in management. This involves trying to educate the patient and the family about the fatigue and how to use body mechanics, energy conservation, in-between breaks, activities and other strategies for minimising fatigue that can be used at home.’ (OT1)*

**Use of antidepressants.** Several DRs mentioned unrecognized treatment such as prescribing antidepressants for patients with PSF to maintain the patient’s energy levels later.

*‘To manage PSF, I think there is a need for antidepressants as they are better for fatigue. The patients can do more practice exercises.’ (DR6)*

*‘The only medications I will consider in such cases PSF are antidepressants.’ (DR1)*

#### 3.2.4. Theme 4: Lack of Awareness about Post-Stroke Fatigue

This theme highlights the limitations imposed by a lack of information and, sometimes, by misdirected information, which hamper the identification and management of PSF.

Almost all the HCPs tended to not address PSF when patients did not complain about it, since stroke survivors did not understand and perceive PSF as a significant problem. It was found that a lack of awareness in this theme did not tie in well with the previous theme (theme 3) where HCPs suggested many treatment approaches.

*‘Patients do not report fatigue, not specifically.’ (DR8)*

*‘They usually don’t talk about fatigue or ask me about it.’ (PT8)*

*“I think most stroke patents believe it’s normal to have fatigue.’ (OT7)*

**Post-stroke fatigue: Not an important health issue**. Some of the participants believed that PSF was not an important health issue, and some of them even acknowledged that they had never discussed PSF and its associated health implications at any scientific seminars or meetings related to stroke.

*‘To be honest, no, it PSF is not something that’s at the top of our most-important list because with a stroke survivor, we would all just go for the physical impairments.’ (OT3)*

*‘Honestly, we have never discussed fatigue after stroke and fatigue as a general topic in our divisional seminars.’ (PT1)*

**Lack of knowledge about post-stroke fatigue among patients’ families.** The physiatrists indicated that patients’ caregivers/families generally lacked knowledge about PSF. Some caregivers were not convinced about the reality of PSF, and this could have adverse consequences for the patient, particularly when families and caregivers began to excessively push the patient to be more active or prevented them from being active in order to protect them. Thus, misunderstandings about PSF among family members and caregivers could undermine patient recovery and overall health.

*‘Most of the time, caregivers don’t believe it PSF. They think that the patient is making excuses not to be active, not to work out, especially when the family is the supportive type. They have high expectations from the patient, or high hopes that the patient will return to normal, and they push the patient extremely.’ (DR1)*

*‘Actually, the caregivers are sometimes overprotective.’ (DR4)*

*‘They (caregiver) are not understanding. They are just trying to push the patient to do more.’ (OT7)*

*‘Usually the stroke patients and caregiver concern about other neurological problem but not the fatigue.’ (PT5)*

#### 3.2.5. Theme 5: Domains for Improvement

**Education and awareness about post-stroke fatigue in healthcare practitioners and patients**. Almost all the participants agreed that substantial improvements were required in the diagnosis and subsequent management of PSF by healthcare practitioners. Most participants, especially the therapists, reported that they had a low level of PSF awareness and needed to learn more about how to evaluate PSF. In addition, almost all the participants felt that educational material for stroke survivors and their caregivers was an area that had received insufficient attention to date.

*‘Maybe we need to ask about the outcome measures of fatigue after stroke, or read about how we can measure it. This is very important.’ (PT5)*

*‘There’s a need to educate the patients and caregivers about PSF. That’s something we don’t pay attention to.’ (PT7)*

**Adequate clinical guidelines for PSF management**. The participants also mentioned the absence of clinical guidelines or inadequate guidelines for PSF management at their work place.

## 4. Discussion

To our knowledge, this qualitative study is the first to explore the perceptions and experiences of HCPs in Saudi Arabia about their understanding and management of PSF. Overall, Saudi HCPs perceived PSF as associated with various demographic, biomedical, and psychological factors. Also, we found that they understood PSF management but this understanding was not reflected in their practice as they emphasized the need for more educational material and tools that could assist them in their practice.

### 4.1. Theme 1: Knowledge about Post-Stroke Fatigue

In the first generated theme, HCPs attributed the incidence and severity of PSF to various biological, demographic, clinical, lifestyle, and psychological factors. Indeed, there is evidence that the aetiology of PSF is multidimensional, as studies have found interactions between PSF and various factors such as age, sex, race, neurological deficits, comorbidities, smoking, drugs, sleep patterns, pain, pre-stroke fatigue level, mood disorders (e.g., depression and anxiety), and cognitive impairment [3,8,9]. Our participants also placed great emphasis on psychological factors. As explained by Ponchel et al. (2015) [2], fatigue is subjective and may be dependent on psychological factors such as stroke-related stress and fear about the consequences of stroke and not being able to return to normal life. Further, fatigue has also been found to be associated with depression [15] and anxiety. However, although there is statistical evidence for the link between these psychological factors (anxiety and depression) and PSF, it is not clearly understood how these symptoms are related to PSF [8,16].

Most participants in our study considered PSF to be related to changes in the endurance levels of stroke survivors. This shows that their understanding of PSF is limited to a single, albeit important, facet of it. Furthermore, a study on the perspectives of HCPs interviewed by [17] about PSF in the UK reported that PSF was not associated with endurance. Contrary to our results, Drummond et al. (2021) [17] found that PSF was not associated with endurance level. However, PSF is a multifaceted condition that involves various biological, psychological, and social factors. Despite the numerous factors associated with fatigue, it is still not clear if they are the root cause of this condition [18].

### 4.2. Theme 2: Diagnosing Post-Stroke Fatigue

According to the second theme described in this study, the participants, in general, used non-specific and non-standardised tools to diagnose and assess PSF. They reported measuring patients’ endurance and functional levels to diagnose and manage PSF. According to Tseng and Marzilli (2018) [19], it is important to first determine the PSF category using a multidimensional model, and then measure the PSF severity level. However, all the current fatigue measurement methods used were designed for other diseases with different causes, such as multiple sclerosis, and were not designed specifically for measuring PSF [9]. Further, it is important to assess and evaluate the patient as objectively as possible [20], but most measurement tools reflect the impact of PSF [21] based on the measurement of various variables. Therefore, there is a need for a clinically approved tool that is scientifically validated for the identification of PSF. This is consistent with the recommendation by English et al. (2023) [18] for the need of a comprehensive assessment for stroke survivors.

### 4.3. Theme 3: Treatment Approaches

The findings in theme three of our study suggest that practices differ between DRs, PTs, and OTs. Despite these differences, all the participants agreed that the confounding factors responsible for the occurrence and progression of PSF needed to be treated to effectively manage PSF. This is a logical and justifiable approach to the management of a stroke survivor, as it involves the treatment of multidimensional factors that can aggravate PSF. This approach is in agreement with that of Hinkle et al. (2017) [9] and the recommendations of physiotherapists in the UK [22]. Another finding of the present study was that many HCPs highlighted the importance of inquiring about sleep and offering advice about improving sleep quality for stroke survivors. In accordance with the HCPs’ approach in our study, sleep disturbance studies on PSF [23] reported that sleep management might alleviate the psychological symptoms accompanying fatigue [24]. The HCPs in our study also used several modes of exercise such as graded aerobic or endurance, breathing, and group exercises which, in turn, reduced fatigue as reported in the previous literature to help stroke survivor patients manage their fatigue levels and reduce fatigue. Graded physical activity and physical exercises, including breathing exercises, can improve both functional and physical outcomes, which, in turn, reduce fatigue [9,25,26].

Another treatment modality that was discussed was energy conservation techniques, such as taking naps, using simplification devices, and resting in between activities, which fell under the recommended treatment modalities [27,28]. Almost all the HCPs in this study discussed the importance of teaching treatment techniques, such as energy conservation, to patients and caregivers to alleviate their fatigue symptoms [27,28]. A cross-sectional survey was conducted for people with PSF in the UK involving 305 HCPs, with the majority being OTs (56%). The survey found that the most used management methods for PSF management were pacing (67%), keeping a fatigue diary and spreading activities throughout the day (39%), and education (38%) [29].

On the other hand, ref. [19] state that there is currently no evidence for effective pharmacological interventions for stroke survivors with PSF. Moreover, in Wu’s (2015) systematic review [30], antidepressants were not found to be effective for the treatment of PSF. Instead, a previous randomized controlled trial [26] demonstrated that cognitive behavioural therapy combined with physiotherapy resulted in a significant improvement in PSF. None of the HCPs in the present study mentioned cognitive behavioural therapy as one of the psychological approaches.

Our above findings related to the various treatment approaches are important, as very few previous qualitative studies have provided such varied perspectives of healthcare practitioners. For example, a qualitative study by Drummond et al. (2021) [17] on PSF reported that the main strategies used were to maintain diaries, to pace activities, to educate patients about fatigue, and to use specific coping strategies. However, most of the sample comprised occupational therapists, and physicians were not included. Thus, the approach was more in line with the perspective of occupational therapists. Further, in a cross-sectional online survey of 71 physiotherapists and 66 occupational therapists, different approaches that focused on both physical and cognitive components were identified within their multidisciplinary teams. However, they did not provide the details in terms of the roles of the members of the team [22]. 

### 4.4. Theme 4: Lack of Awareness about Post-Stroke Fatigue

A lack of awareness about PSF among stroke survivors and their caregivers and among HCPs themselves was the fourth theme of this study. Several participants mentioned that patients were neither asked about their fatigue symptoms nor complained directly about these during consultations or therapy sessions. For the delivery of successful individualised care, good and effective communication between HCPs and patients is essential [31]. According to Kennedy and Kidd (2018) [32], ignorance about PSF among healthcare practitioners, patients, and caregivers, coupled with the patients’ inability to adequately express its symptoms, results in this condition being missed in a significant number of stroke survivors undergoing rehabilitation. However, there is a great deal of uncertainty about how PSF is identified and managed as part of wider stroke rehabilitation practice, and there are currently no standard guidelines or criteria for the diagnosis or treatment of PSF [33]. A focus group study was conducted to explore the experiences and perceptions of stroke survivors and health professionals regarding PSF guidance in Dutch rehabilitation and follow-up care. The study found that providing fatigue guidance, such as tailored advice on pacing activities during and after stroke rehabilitation, could fulfil the current unmet need of stroke patients for coping with fatigue to improve participation, physical activity, and overall health. Additionally, providing information at the optimal time and extensive follow-up support for individuals after stroke rehabilitation may help to ensure that the right people receive the right care at the right time [34].

### 4.5. Theme 5: Domains for Improvement

This theme sheds light on participants’ recommendations for improving the treatment and management of PSF. The participants mentioned the importance of including PSF in their educational agenda, specifically, knowledge about the correct method of conducting and performing the PSF evaluation and devising an appropriate treatment plan for the management of PSF. To help improve the quality of life for stroke survivors by reducing the impact of PSF, several updated guidelines globally have provided suggestions based on consensus. However, these clinical guidelines do not provide strong recommendations for the management of PSF [18].

This study was the first in Saudi Arabia to conduct in-depth interviews with HCPs actively involved in managing stroke survivors with PSF. The inputs of the HCPs in this study will help deepen the understanding of PSF and its treatment approaches and aid future policymaking in Saudi Arabia. They will also stimulate further research on the topic that can add to the current knowledge about PSF. A major limitation of this study is that it was carried out at only one rehabilitation centre in Saudi Arabia. However, the selected institution is a tertiary care centre of excellence where patients from all over the country are being treated. Future studies including participants from multiple centres across different regions to enhance the study’s generalizability is recommended. Also, the primary researcher is a physiotherapist and this could have introduced bias into the study related to data collection. However, this was minimised by adopting a semi-structured interview format, thereby keeping the researcher’s interactions with the participants structured. Another limitation is that this study has not considered the stroke stages when interviewing the HCPs. Also, not all other HCPs, who may have different contextual perceptions, were included in this study, such as nurses, speech and language therapists, and dieticians. This might hinder the potential richness of the data collected. Therefore, a wider range of HCPs is recommended to be involved in future PSF studies. Also, more professional training about PSF among HCPs and education among stroke survivors and caregivers/families in Saudi Arabia is needed.

## 5. Conclusions

In conclusion, the concept of PSF, its associated factors, and its management are understood by HCPs in Saudi Arabia, but this understanding is not reflected in their practice. This was evident in how they managed PSF using the treatment methods they were familiar with and the lack of PSF-related information in the educational materials provided to their stroke survivor patients. It seems that HCPs also still use non-standardized tools for assessing PSF, even though previous studies have recommended using designed specific assessments to determine the category and measure the severity level of PSF. As there are no high-quality evidence-based guidelines for PSF management, this study suggests various treatment approaches, including the management of confounding factors that could aggravate PSF symptoms, sleep management exercises, and energy conservation techniques. Finally, the findings indicated that there was consensus among the interviewed HCPs about the need to improve awareness about PSF among healthcare practitioners, stroke survivors, and their caregivers/families.

The practical implications based on this study’s findings include:

1. Professional training for HCPs about tools for diagnosing and managing PSF is strongly recommended.
2. PSF-specific assessment tools in the clinical routine for stroke survivors need to be utilised by HCPs.
3. Education among stroke survivors and caregivers/families in Saudi Arabia is needed.
4. The inputs of the HCPs in this study will help deepen the understanding of PSF and its treatment approaches in clinical practice.

## Figures and Tables

**Table 1:** Interview guide.

Interview topic guide question ❖ Questions for physiatrist, physiotherapist, and occupational therapists: Can you tell me briefly about your background and expertise in relation to post-stroke fatigue? Do your stroke survivors describe their fatigue after stroke during sessions? Do they describe it in the same way or are there different experiences of post-stroke fatigue? If so, how? Are you aware of post-stroke fatigue as an issue? If so, where did you learn about this? (continuous professional development activities internal to your department, meetings, conferences, and journal club) Can you think of ways in which you have helped people deal with post-stroke fatigue? (any management plan or intervention you currently provide for post-stroke fatigue to your patients, or any intervention you think has helped post-stroke fatigue patients to treat or reduce fatigue symptoms after stroke) How did family/caregivers respond to the fatigue stroke survivors? Did they understand it? And could you explain their response in more detail? Do you think there is a need to improve your current practice around post-stroke fatigue? If yes, how do you think you could improve your practice around it? Would you like to add anything else we might have missed in the talk above?

**Table 2:** Themes and subthemes generated from the data analysis.

Themes	Subthemes and Sub-Subthemes
1—Knowledge about post-stroke fatigue	1.1 Demographic, clinical, and lifestyle factors 1.2 Psychological factors
2—Diagnosing post-stroke fatigue	2.1 Assessing endurance levels 2.2 Assessing functional levels
3—Treatment approach	3.1 Managing confounding factors 3.2 Sleep management 3.3 Exercise therapy 3.3.1 Endurance and breathing exercises 3.3.2 Group exercises 3.4 Energy conservation techniques 3.5 Education of patients and caregivers 3.6 Use of anti-depressants
4—Lack of awareness about post-stroke fatigue	4.1 Post-stroke fatigue: Not an important health issue 4.2 Lack of knowledge about post-stroke fatigue among patients’ families
5—Domains for improvement	5.1 Education and awareness about post-stroke fatigue in healthcare practitioners and patients

## Data Availability

The datasets analyzed during the current study are available from the principal author on reasonable request.
